# Short-Term Intake of *Euphorbia tirucalli* Latex Modifies Kidney Function in Rats: Possible Role of Oxidative Stress and Inflammatory Response

**DOI:** 10.3390/antiox14070856

**Published:** 2025-07-13

**Authors:** Edgar Hell Kampke, Maria Eduarda Souza Barroso, Leonardo da Silva Escouto, Luciana Polaco Covre, Ágata Lages Gava, Bianca Prandi Campagnaro, Ricardo Machado Kuster, Silvana Santos Meyrelles

**Affiliations:** 1 Graduate Program in Physiological Sciences, Federal University of Espirito Santo, Av. Mal. Campos, 1468, Maruipe, Vitoria 29047-105, ES, Brazil; edkampke@hotmail.com (E.H.K.); m.eduarda.sb@live.com (M.E.S.B.); leonardo_lse@hotmail.com (L.d.S.E.); agata.gava@ufes.br (Á.L.G.); 2 Division of Medicine, University College London, London WC1E 6BT, UK; luciana.covre@ufes.br; 3 Infectious Diseases Center, Federal University of Espirito Santo, Vitoria 29047-100, ES, Brazil; 4 Graduate Program in Pharmaceutical Sciences, Vila Velha University (UVV), Vila Velha 29102-920, ES, Brazil; bianca.campagnaro@uvv.br; 5 Department of Chemistry, Federal University of Espirito Santo, Vitoria 29047-105, ES, Brazil; ricardo.m.kuster@ufes.br

**Keywords:** *Euphorbia tirucalli*, renal function, mean arterial pressure, oxidative stress, inflammatory activity

## Abstract

Medicinal plants have been traditionally used for generations, often without scientific validation. *Euphorbia tirucalli* (*E. tirucalli*), a plant native to Africa, is commonly employed in folk medicine for treating various ailments, including cancer. However, most studies involving this species are limited to in vitro models, and its systemic effects remain poorly understood. This study aimed to evaluate the impact of *E. tirucalli* latex on renal function in healthy Wistar rats. Animals were divided into two groups: a control group receiving water and a treated group receiving *E. tirucalli* latex (13.47 mg/kg) by gavage for 15 days. Renal function was assessed by measuring glomerular filtration rate (GFR), renal plasma flow (RPF), renal blood flow (RBF), renal vascular resistance (RVR), and mean arterial pressure (MAP). Additionally, oxidative stress markers, reactive oxygen/nitrogen species, and inflammatory activity were analyzed in renal tissue. *E. tirucalli* significantly reduced GFR, RPF, and RBF, while increasing RVR and MAP. Renal tissue exhibited elevated levels of advanced oxidation protein products, myeloperoxidase, nitric oxide, and peroxynitrite/hydroxyl radicals. These findings indicate that *E. tirucalli* latex adversely affects renal hemodynamics and promotes oxidative and inflammatory damage, suggesting potential nephrotoxic effects, even in healthy subjects.

## 1. Introduction

The use of natural products, especially plants, in the form of infusions, decoctions, or solutions, has been a worldwide practice for relieving symptoms or treating a variety of diseases. Medical plants are commonly used for gastrointestinal disorders, diabetes, hypertension, anxiety, and cardiovascular conditions [1]. Among these, is *Euphorbia tirucalli* Linn. (*E. tirucalli*), popularly known as Aveloz. It belongs to the Euphorbiaceae family, it originates from Africa, and has achieved a broad geographic distribution, including Brazil, where favorable climatic conditions support its growth [2,3,4]. The latex produced by this plant is widely used by populations in northeastern Brazil for its reported laxative, antimicrobial, and antiparasitic effects, and it is traditionally employed to treat conditions such as asthma, cough, earache, rheumatism, warts, cancer, skin tumors, and syphilis [5,6].

*Euphorbia tirucalli* latex contains several bioactive compounds, including the triterpenes euphol and tirucallol, and the diterpenes phorbol esters (e.g., 4-deoxyphorbol, forbol) and ingenol derivatives, which are considered the most pharmacologically relevant constituents. While triterpenes are primarily associated with anti-inflammatory and therapeutic effects [7], the diterpenes—particularly phorbol esters—are regarded as toxic and potentially tumor-promoting, due to their ability to activate protein kinase C (PKC) [8,9,10]. The chemical composition of the latex may vary, depending on its state: fresh latex contains isoeuphorol and tirucallol, whereas dried latex is characterized by the presence of euphorone and resin [6,8].

Based on its popular use, numerous studies have been conducted to identify the therapeutic properties of *E. tirucalli*. Researchers have demonstrated that although the latex possesses caustic and corrosive properties, causing conjunctivitis and skin dermatitis [11], it can also exhibit significant biological activities, such as antinociception [12] and angiogenic effects [13]. Additionally, it has been found to possess cytotoxic properties [14] and has the capacity to reduce tumor growth and cachexia, and induce immunomodulation [15]. However, evidence also suggests that latex or its compounds exhibit pro-inflammatory activity [16], including an increase in the expression of cytokines such as TNF-α and IFN-γ [17].

Despite these findings, most studies to date have been conducted in vitro, and little is known about the systemic effects of *E. tirucalli* latex when ingested, particularly on vital physiological systems such as the kidneys. Furthermore, the indiscriminate use of this latex by the population—especially for its supposed antitumoral properties—raises concerns about its potential toxicity.

Therefore, this study aims to fill this gap by investigating the short-term effects of *E. tirucalli* latex ingestion on renal function, oxidative stress, and inflammatory markers in normotensive rats. By addressing this underexplored area, we hope to contribute to a better understanding of the biological impact and safety profile of this widely used medicinal plant.

## 2. Materials and Methods

### 2.1. Plant Material Preparation and Extraction

*E. tirucalli* latex was collected on 3 November 2019 at 09:00 a.m. in Vila Velha city, ES, Brazil (−20.3778279° S, −40.3063419° W), using aerial parts (tubers) of uniform size from a single plant to ensure consistency and reduce potential variability in chemical composition. Six drops of latex (13.47 mg/kg) were added to microtubes pre-filled with 500 μL of water. The microtubes were stored at 4 °C until use. A plant specimen was submitted to botanical identification and stored under registration RFA31675. To determine the administered dose, we first measured the physicochemical characteristics of the latex. The average volume per drop was calculated from 10 replicates (20 μL/drop), and the density was determined by using a pycnometer (1.0866 g/mL). The dosage of 13.47 mg/kg was calculated based on traditional human usage of *E. tirucalli* latex in Brazil. In the late 1960s, physician Lauro Neiva prescribed a regimen of 6 drops of pure latex diluted in water for 3 consecutive days for patients with cancer and Chagas disease, a practice still followed today [18,19]. To translate this human dosage into an equivalent rat dose, we followed the FDA guidelines for body surface area (BSA)-based dose conversion [20,21]. Using Equation (1), and assuming a 60 kg human and the respective Km factors (6.2 for rats; 37 for humans), the human dose of 6 drops (0.1 drops/kg) translates to approximately 0.62 drops/kg in rats. Multiplying this by the average volume and applying the latex density yielded a final dose of 13.47 mg/kg.

(1) $$Human\;Equivalent\;Dose\left(\frac{drops}{kg}\right)=Animal\;Dose\left(\frac{drops}{kg}\right)\times \frac{Km\;animal}{Km\;human}$$

All experimental procedures described below were conducted at the Laboratory of Integrative Physiology within the Graduate Program in Physiological Sciences at UFES (Vitória, Brazil), except for the flow cytometry analyses, which were carried out at the Laboratory of Molecular and Cellular Immunology, Center for Infectious Diseases (UFES, Vitória, Brazil).

### 2.2. Experimental Animals and Study Design

Experiments were conducted using six-month-old male Wistar rats obtained from the animal facilities of the Health Sciences Center at the Federal University of Espirito Santo. Rats received a normal chow diet and had access to water ad libitum. They were housed in temperature-controlled rooms (22 °C) under a 12:12 h light–dark cycle. All experimental procedures were performed following the National Council for Animal Experimentation Control Guide (CONCEA) for the Care and Use of Laboratory Animals, and the protocols were previously approved by the Institutional Ethics Committee for the Use of Animals (CEUA-UFES, protocol no. 01/2022, approval date: 10 July 2022).

Rats were randomly allocated into two groups (n = 8 per group). The *E. tirucalli* group received *Euphorbia tirucalli* latex (13.47 mg kg^−1^) freshly diluted each day in 1.0 mL of sterile distilled water using a 1.5 mL polypropylene microtube; the full volume was administered by oral gavage for 15 days. The control group received the same volume (1.0 mL) of the identical vehicle—sterile distilled water prepared in an identical 1.5 mL polypropylene microtube—and this was also administered by oral gavage for 15 days. Thus, the vehicle was identical in both groups, differing only in the presence or absence of latex.

After the treatment period, the animals were anesthetized with thiopental sodium (50 mg/kg, i.p.) to evaluate mean arterial pressure (MAP) and renal function. After this, biological materials such as kidneys and tibia were removed for kidney/tibia ratio, oxidative stress, inflammation, reactive oxygen species (ROS), and reactive nitrogen species (RNS) levels analysis.

### 2.3. MAP and Renal Function Measurements

The animals were anesthetized with sodium thiopental (50 mg/kg, i.p.), and a tracheostomy was performed to facilitate breathing. Polyethylene catheters were inserted into the femoral artery for mean arterial pressure (MAP) measurement and into the femoral vein for drug administration and blood sample collection. A catheter was also placed in the bladder to allow for urine collection. The arterial catheter was connected to a pressure transducer (Cobe Laboratories, Lakewood, CO, USA), which was linked to a pressure-processor amplifier and a data acquisition system (MP100, Biopac Systems, Goleta, CA, USA) for continuous monitoring of arterial blood pressure.

Renal function was assessed using inulin and sodium para-aminohippurate (PAH) clearances to estimate glomerular filtration rate (GFR) and renal plasma flow (RPF), respectively [22]. After a 30 min stabilization period, the animals received a saline infusion containing 3% mannitol at a rate of 0.06 mL/min. This was followed by a bolus injection of 1 mL saline containing inulin (300 mg/kg) and PAH (6.66 mg/kg). Subsequently, a continuous infusion of a solution containing saline, inulin (15 mg/mL), PAH (4 mg/mL), and 3% mannitol was maintained throughout the experiment. Four urine samples and four corresponding blood samples were collected at 30 min intervals.

Hematocrit was measured using heparinized capillary tubes at each collection time point. Inulin and PAH concentrations in plasma and urine were determined using a colorimetric assay [23]. Renal blood flow (RBF) and renal vascular resistance (RVR) were calculated using the following equations:

(2) $$RBF=\frac{RPF}{\left(1-Hematocrit\right)}$$

(3) $$RVR=\frac{MAP}{RBF}$$

where

RBF = renal blood flow (mL/min)
RPF = renal plasma flow (mL/min)
Hematocrit = fraction of blood volume occupied by red blood cells
RVR = renal vascular resistance (mmHg·min/mL)
MAP = mean arterial pressure (mmHg)

### 2.4. Kidney Morphometric Measurement

After evaluating renal function and MAP levels, the animals were euthanized with a sodium thiopental overdose (100 mg/kg, i.p.). The kidneys and tibias were then removed and washed in a saline solution. The ratio of kidney weight (mg) to tibia length (cm) was calculated and used as a renal hypertrophy index [24].

### 2.5. Kidney Oxidative Stress Analysis

Renal tissue homogenate was prepared after renal function experiments. Briefly, kidneys were collected, finely triturated with surgical scissors, and ground at high speed with a tissue homogenizer (Ultra 380—Ultra Stirrer—Round Rock, TX, USA). This process was carried out for approximately 15 min at 37 °C, resulting in a final solution of cells and phosphate-buffered saline (PBS). The samples were then stored at −80 °C, until further use.

Total protein was quantified using the colorimetric method using Bradford’s reagent as a chromogenic reagent at a wavelength of 595 nm [25] using a spectrophotometer microplate reader (Sinergy H1—BioTek^®^—Winooski, VT, USA). Kidney oxidative stress levels were determined by quantifying Advanced Oxidation Protein Products (AOPPs) using a colorimetric method with chloramine T. Briefly, 40 μL of renal tissue homogenate dilution (1:5 in PBS) was mixed with 10 μL of 1.16 M potassium iodide solution and 20 μL of glacial acetic acid in a 96-well microplate. The absorbance was measured at a wavelength of 340 nm, and the results are expressed in μM chloramine T equivalents [26]. This result represents the ratio between the concentration of oxidized proteins and the total protein concentration.

### 2.6. Kidney Inflammatory Activity Measurement

The presence of inflammation in the renal cells was determined through myeloperoxidase (MPO) activity levels, as previously described [27]. In brief, twelve microliters of renal tissue homogenate were combined with 236 µL of 50 mM phosphate buffer, pH 6.0, containing 0.167 mg/mL o-dianisidine dihydrochloride and 0.0005% hydrogen peroxide. The absorbance was read at a wavelength of 460 nm at 15 s intervals for 10 min. The results are expressed as units of myeloperoxidase activity (a.u. myeloperoxidase) over time.

### 2.7. Kidney Cell Isolation for ROS and RNS Evaluation

Enriched kidney cell fractions from the experimental groups were obtained based on previous studies [28,29]. In brief, following the renal function protocol, kidneys were removed and finely triturated with surgical scissors, followed by incubation with an extraction solution containing trypsin (Sigma-Aldrich, St. Louis, MO, USA) to dissociate the cells. The cell extract was then filtered through a nylon screen (BD Falcon 70 μm) to remove cell debris. The samples were washed twice with phosphate-buffered saline (PBS) and stored at −80 °C until further analysis.

### 2.8. Quantification of Kidney Cells ROS and RNS Production

Quantification of intracytoplasmic ROS and RNS components was conducted using flow cytometry with an FACSCanto II instrument (Becton Dickinson, BD, San Diego, CA, USA), as previously described [28,30]. Superoxide anion (O_2_^·−^), hydrogen peroxide (H_2_O_2_), peroxynitrite/hydroxyl radical (ONOO^·−^/·OH^−^), and nitric oxide (·NO) were monitored separately by measuring changes in median fluorescence intensity (MFI) emitted by dihydroethidine (DHE), dichlorofluorescein (DCF), hydroxyphenyl fluorescein (HPF), and diaminofluorescein (DAF) probes, respectively. Briefly, 1 × 10^6^ cells were incubated with 160  µM DHE, 20  mM DCF, 10 μM HPF, or 2  μM DAF at 37 °C for 30 min (DHE, DCF, and HPF) or 180 min (DAF) in the dark. The samples were then washed, resuspended in PBS, and kept on ice until the acquisition of 10,000 events by flow cytometry. Subsequently, the data were analyzed using FlowJo X 10.0.7r2 software (Becton Dickinson, BD, San Diego, CA, USA) and data were expressed as Median fluorescence intensity in arbitrary units (MFI a.u.).

### 2.9. Data Analysis

All data are presented as the mean ± SEM for each group. The normal distribution of data was assessed using the Kolmogorov–Smirnov test. As all the samples exhibited a Gaussian distribution, an unpaired Student’s *t*-test was employed for statistical analysis following the administration of *E. tirucalli*. *p*-values less than 0.05 (*p* < 0.05) were considered statistically significant. The statistical analysis was performed using GraphPad Prism version 8.02 (GraphPad Software, San Diego, CA, USA).

## 3. Results

### 3.1. Effects of Aveloz Treatment on MAP Levels and Renal Function

**Figure 1:**
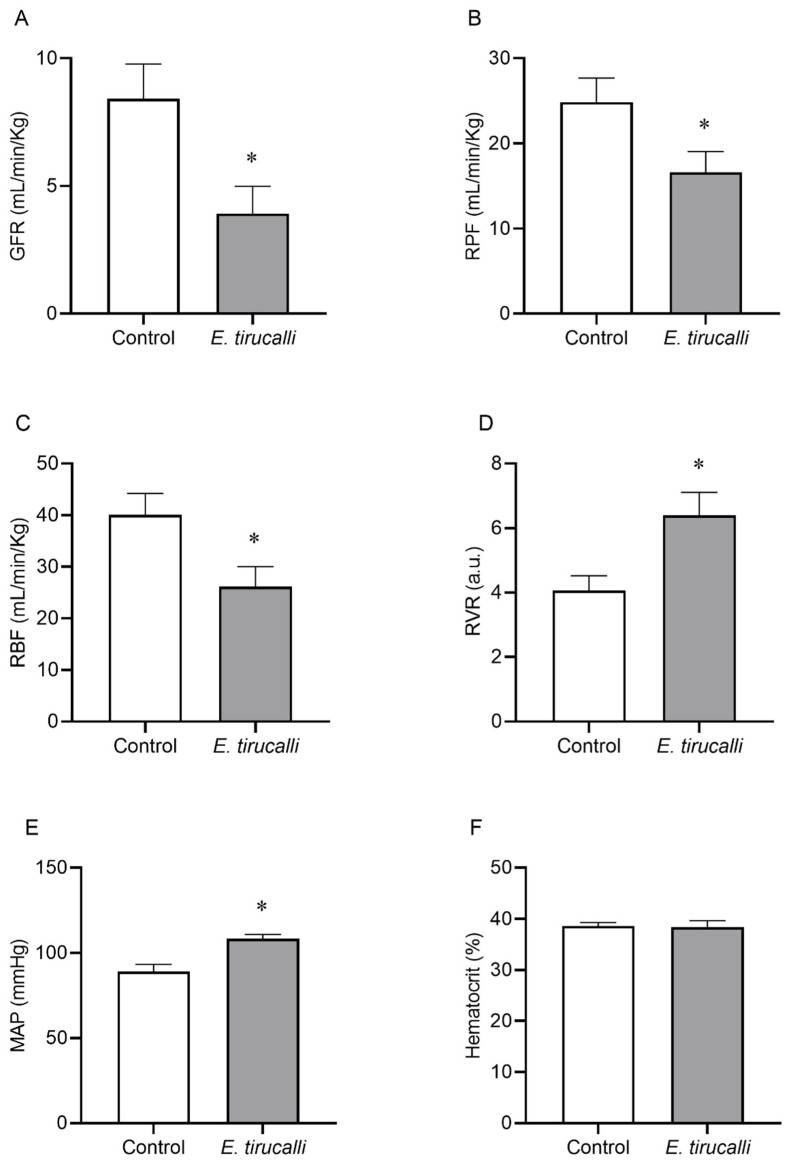
Renal Hemodynamics. The graphs show the results for glomerular filtration rate (GFR, (**A**)), renal plasma flow (RPF, (**B**)), renal blood flow (RBF, (**C**)), renal vascular resistance (RVR, (**D**)), mean arterial pressure (MAP, (**E**)) and hematocrit percentage (**F**) in animals treated with vehicle (white bars, n = 8) or *E. tirucalli* latex for 15 days (gray bars, n = 8). Data are presented as mean ± SEM. The asterisk indicates the significance level of the post hoc comparison between groups (* *p* < 0.05 vs. control group).

Figure 1A–F shows that Aveloz treatment significantly decreases the GFR (3.9 ± 1.1 mL/min/kg vs. 8.4 ± 1.3 mL/min/kg), RPF (16.66 ± 2.8 mL/min/kg vs. 24.7 ± 2.8 mL/min/kg), and the RBF (26.14 ± 3.8 mL/min/kg vs. 40.1 ± 4.1 mL/min/kg) levels, respectively, in the *E. tirucalli* group, compared to the control group. Interestingly, statistically significant changes were observed in RVR (6.4 ± 0.7 a.u. vs. 4 ± 0.4 a.u.) values and MAP levels (108.4 ± 2.5 mmHg vs. 89 ± 4.2 mmHg) in the *E. tirucalli* group, compared to the control group, respectively. No significant differences were found between hematocrit values between the *E. tirucalli* and control animals (38.38 ± 1.28% vs. 38.57 ± 0.68%), respectively.

### 3.2. Kidney/Tibia Ratio Analysis

Aveloz treatment for 15 days did not change the kidney/tibia ratio compared to the control group (Table 1).

### 3.3. Renal Oxidative Stress and Inflammatory Response

**Figure 2:**
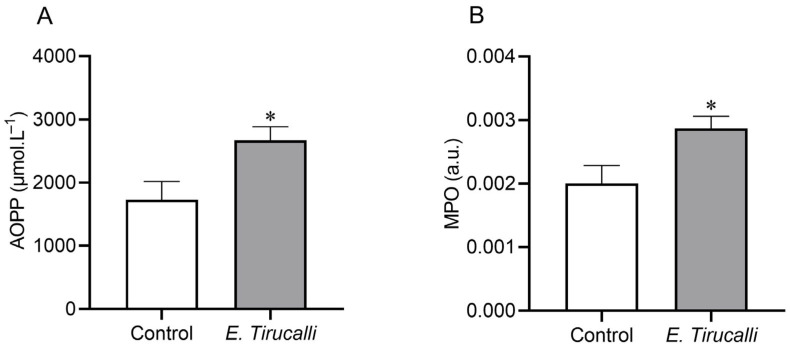
Oxidative stress measurement. Kidney protein oxidation measured through advanced oxidation protein product (AOPP) levels (**A**) and kidney inflammation measured through myeloperoxidase (MPO) activity (**B**) in control animals (white bars, n = 8) and *E. tirucalli*-treated animals (gray bars, n = 8). * *p* < 0.05 vs. control group. Data are presented as mean ± SEM. The asterisk indicates the significance level of post hoc comparison between groups (* *p* < 0.05 vs. control group).

Figure 2A shows that the analysis of the AOPP protein oxidation marker revealed that *E. tirucalli* latex ingestion resulted in a statistically significant increase in kidney oxidative stress production in the treated group, compared to control animals (2.670 ± 215.4 μmol·L^−1^ vs. 1.729 ± 289.2 μmol·L^−1^, *p* < 0.05, respectively). Additionally, to investigate whether *E. tirucalli* latex could induce renal inflammation, we measured the MPO enzyme activity and found, as shown in Figure 2B, that treated animals exhibited significantly increased MPO activity levels of this inflammatory marker compared to control animals (0.002868 ± 0.0001934 a.u. vs. 0.001999 ± 0.0002845 a.u., *p* < 0.05, respectively).

### 3.4. Kidney Cells ROS and RNS Production

**Figure 3:**
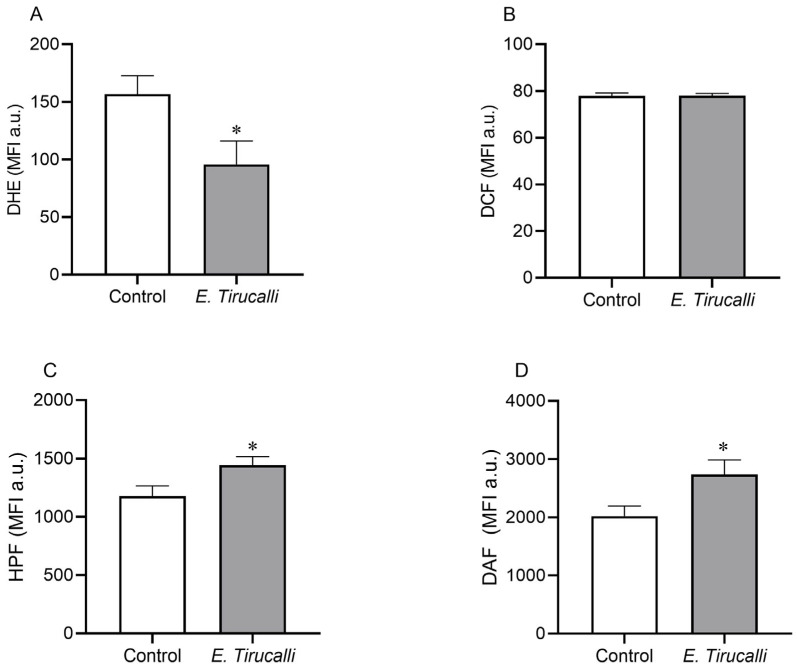
ROS and RNS levels. Kidney intracytoplasmic production of superoxide anion (**A**), hydrogen peroxide (**B**), nitric oxide (**C**) and peroxynitrite and hydroxyl radical (**D**) production, measured by DHE, DCF, DAF and HPF fluorescence probes, respectively, in control animals (white bars, n = 8) and *E. tirucalli*-treated animals (gray bars, n = 8). *p < 0.05 vs. control group. Data are presented as mean ± SEM from median fluorescence intensity in arbitrary units (MFI a.u.). The asterisk indicates the significance level of post hoc comparison between groups (* *p* < 0.05 vs. control group).

The intracytoplasmic production of ROS and RNS in kidney cells was measured using a flow cytometer with DHE, DCF, HPF, and DAF fluorescent probes, as shown in Figure 3A–D. *E. tirucalli* latex-treated animals exhibited a significant decrease in superoxide anion levels compared to the control group (95.78 ± 20.31 MFI a.u. vs. 156.7 ± 15.86 MFI a.u., *p* < 0.05, Figure 3A). In contrast, the production of both peroxynitrite/hydroxyl (1.442 ± 74.70 MFI a.u. vs. 1.176 ± 90.45 MFI a.u.) and nitric oxide (2.737 ± 249.6 MFI a.u. vs. 2.020 ± 174.1 MFI a.u.) was statistically significantly increased in the *E. tirucalli* group compared to the control group (*p* < 0.05, respectively). No significant differences in hydrogen peroxide production were observed between the treated and control groups (78.05 ± 0.89 MFI a.u. vs. 77.93 ± 1.21 MFI a.u., respectively).

## 4. Discussion

The widespread use of *E. tirucalli* within the Brazilian population contrasts with the limited understanding of its therapeutic potential and interactions with physiological systems. Given that plants contain diverse bioactive metabolites with the capacity to induce toxicity in various organs and systems [31], uncertainties persist regarding the therapeutic and toxicological effects of *E. tirucalli* treatment, particularly concerning its impact on different organs and systems. Consequently, a significant scientific gap remains regarding both the therapeutic efficacy and the potential for renal toxicity associated with *E. tirucalli* ingestion in healthy individuals.

While endogenous markers such as creatinine and urea are commonly employed to assess renal function, due to their ease of measurement [32], our study adopted a novel approach by utilizing more precise and reliable methods and markers to evaluate the influence of *E. tirucalli* on the renal system. Despite being more invasive, the analysis of inulin clearance offers superior accuracy in determining GFR, as inulin undergoes complete filtration without reabsorption or tubular secretion. Furthermore, parameters like renal plasma flow, which cannot be accurately assessed with conventional biochemical markers, were reliably measured through the infusion of para-aminohippuric acid (PAH), given its high rate of renal clearance via filtration and secretion [33].

Employing these precise markers, our analysis of renal function revealed that *E. tirucalli* treatment, consistent with its popular use, induced a significant decrease in GFR, RPF, and RBF, while concurrently elevating RVR and MAP. The delicate balance of vascular resistance in afferent and efferent arterioles is critical for regulating RBF and GFR [34]. The significant increase in RVR observed in our study appears to be influenced by alterations in RBF and MAP. Further investigation indicated that the reduction in RPF, with hematocrit remaining stable, likely contributed to the decreased RBF values. Additionally, the significant increase in MAP theoretically explains the observed rise in RVR.

It is important to consider that the observed changes in renal blood flow (RBF) and mean arterial pressure (MAP) may be influenced by factors beyond the direct renal effects of *E. tirucalli* latex. Although all animals were maintained under consistent anesthesia and fluid management, the use of thiopental—known for its dose-dependent cardiovascular depressant effects—may have contributed to systemic hemodynamic alterations, including hypotension and reduced renal perfusion [35,36]. Additionally, even mild or subclinical dehydration can influence RBF and MAP through activation of the renin–angiotensin–aldosterone system and alterations in plasma osmolality [37]. Moreover, systemic oxidative stress has been shown to impair endothelial function and vascular tone, potentially affecting renal perfusion, independently of local oxidative injury [38]. While our experimental design aimed to minimize these confounding factors by standardizing anesthesia protocols, maintaining hydration, and controlling temperature, their potential contribution to the hemodynamic outcomes observed cannot be completely excluded. Therefore, further studies assessing systemic redox status, hydration state, and the effects of alternative anesthetic agents are warranted, to better elucidate the specific role of *E. tirucalli* latex in mediating these changes.

Normally, changes in RVR, driven by alterations in MAP and RBF (influenced by RPF), are counteracted by myogenic mechanisms, to maintain a stable GFR. However, our findings suggest that the impairment of renal function induced by *E. tirucalli* renders these myogenic regulatory mechanisms insufficient, leading to a reduction in GFR. Notably, the increase in RVR due to vascular constriction, coupled with the myogenic response to elevated blood pressure, may also be influenced by phorbol esters present in *E. tirucalli*, which are known to affect renal vasculature by stimulating PKC in vascular tissue, resulting in vasoconstriction [39,40].

Given the scarcity of data on the effects of *E. tirucalli* on renal function, our findings gain importance when compared with studies on other species within the same genus. The reported nephrotoxic potential of *Euphorbia paralias*, particularly in individuals with a history of nephrotic syndrome [41], contrasts with a study showing a nephroprotective effect of *Euphorbia paralias* pretreatment in a kidney injury model, possibly mediated by its antioxidant activity [42]. Thus, our innovative study uniquely demonstrates that the popular use of *E. tirucalli* significantly alters key parameters of renal function regulation.

Renal hypertrophy is a significant adaptive response to sustained alterations in renal function. Under physiological conditions, a balance between protein synthesis and degradation maintains cell size. Increased kidney size often reflects hypertrophy in glomerular and tubular cells, resulting from increased protein synthesis and, consequently, increased cell size and total protein content [43]. The kidney/tibia ratio is a well-accepted index for renal hypertrophy, as changes in renal tissue mass indicate alterations in cellular composition, and tibial length normalizes for individual size differences, independently of body mass changes [43,44].

Our study demonstrated that the renal hypertrophy index was not affected by *E. tirucalli* treatment. While studies with similar objectives have shown varied results, including a reduction in thioacetamide-induced renal hypertrophy with *Euphorbia paralias* treatment (where renal tissue mass was normalized by body weight) [42], our findings indicate that *E. tirucalli* treatment induces significant changes in renal function without causing structural alterations in renal cell size over the 15-day period.

We hypothesize that the *E. tirucalli* treatment, beyond altering renal function parameters, may also influence biomolecular parameters that could potentially lead to severe functional disorders in the long term [45], with oxidative stress being a prime candidate for investigation. Oxidative stress is a well-established factor in kidney disorders and alterations in biomolecular markers [45,46]. The kidney’s high metabolic activity, receiving approximately 25% of cardiac output and possessing a dense mitochondrial population, makes it particularly susceptible to reactive oxygen species (ROS) production under stress conditions [47,48,49].

Our in situ analysis revealed elevated levels of protein oxidation (AOPP) in the *E. tirucalli* group, indicating that ROS influenced renal tissue structure and function [50]. This finding is noteworthy, as there are limited reports correlating oxidative stress, proteins, and the use of plants like *E. tirucalli*, highlighting the novelty of our research. Our observation of increased AOPP aligns with a study on *Euphorbia bicolor* latex [51], suggesting a pro-oxidant effect of *Euphorbia* genus latex, contrary to some literature. The increased MPO enzyme activity in our treated group further indicates pro-inflammatory activity associated with oxidative stress, supporting the involvement of oxidative stress-induced changes in renal function alterations. Notably, our study uniquely links inflammation and oxidative stress as a consequence of *E. tirucalli* use, contrasting with a report of antioxidant-mediated MPO reduction by another *Euphorbiaceae* member [52], underscoring the distinct pro-oxidant nature of *E. tirucalli*.

To further elucidate the effects of *E. tirucalli* on oxidative stress, we quantified ROS and RNS production at the cellular level. Nitric oxide (NO), a crucial regulator of renal hemodynamics, can interact with ROS to form damaging peroxynitrite [53]. Our data revealed a significant reduction in superoxide anion (O_2_^·−^) levels in the treated group, while hydrogen peroxide (H_2_O_2_) levels remained unchanged. Conversely, we observed a significant increase in both nitric oxide (NO·) and peroxynitrite levels in *E. tirucalli*-treated animals. We hypothesize that the reduction in O_2_^−^ levels may be due to its interaction with the increased NO·, leading to peroxynitrite formation [54]. The stable H_2_O_2_ levels, despite reduced O_2_^·−^, might be explained by a potential decrease in SOD expression induced by oxidative stress [55], redirecting O_2_^·−^ towards NO· reaction. It is important to highlight that our study provides novel quantification of ROS and RNS as a consequence of *E. tirucalli* treatment, a methodology routinely employed by our group [56], with limited application to other Euphorbiaceae species. A study on *Euphorbia bicolor* latex reported increased H_2_O_2_ in trigeminal cells [51], contrasting with our findings and potentially reflecting differences in cell types and NO· influence.

The observed alterations in renal function and the marked increase in oxidative stress—particularly reactive nitrogen species (RNS) such as nitric oxide (NO·) and peroxynitrite—suggest that *E. tirucalli* latex, even with short-term dosages, exerts significant biological effects. We hypothesize that this oxidative environment may trigger signaling pathways in renal cells, including the activation of protein kinase C (PKC). Phorbol esters—bioactive compounds identified in *E. tirucalli*—are well-known PKC activators, especially of conventional isoforms such as PKC-α and PKC-β, and have been widely used as pharmacological tools to activate PKC in various tissues [57,58]. PKC activation has also been implicated in oxidative stress processes, acting either as a cause or consequence, with antioxidant compounds shown to modulate PKC activity [59,60,61]. Moreover, pro-inflammatory mediators like arachidonic acid, which are elevated under oxidative conditions, can further stimulate PKC signaling pathways [62]. Given the established role of PKC isoforms in renal diseases—particularly in modulating oxidative and inflammatory responses [63,64]—we propose that PKC activation, mediated by both phorbol esters and oxidative stress, may contribute to the renal dysfunction observed in our study [65]. Although we did not directly assess the expression or activation of PKC isoforms in renal tissue, this remains an important avenue for future research, to clarify isoform-specific roles in *E. tirucalli*-induced renal injury. Further elucidation of these molecular pathways will be crucial to understand the precise mechanisms underlying the nephrotoxic effects observed, guiding safer therapeutic applications or cautionary measures regarding *E. tirucalli* use.

## 5. Conclusions

In conclusion, the findings of this study provide compelling evidence that short-term oral administration of *Euphorbia tirucalli* latex (15 days) can significantly alter renal function parameters—including GFR, RPF, RBF, RVR, and MAP—as well as promote oxidative stress and inflammatory activity in renal tissue. These alterations are likely mediated by bioactive constituents such as phorbol esters, known activators of protein kinase C (PKC), which can trigger oxidative and inflammatory pathways.

To our knowledge, this is the first study to characterize the systemic physiological effects of *E. tirucalli* latex ingestion on renal function in vivo. The results highlight the importance of critically reassessing the popular perception of safety associated with traditional plant-based remedies. Given the observed adverse effects, our data reinforces the need for caution regarding the indiscriminate use of *E. tirucalli*, particularly in the absence of rigorous toxicological and pharmacological evaluation.

However, despite the relevant findings, the 15-day treatment period represents a relatively short timeframe, and it remains unclear whether the observed renal alterations are reversible, progressive, or might stabilize over time. Further studies are warranted to determine the dose–response relationship, long-term effects, and the reversibility or progression of renal alterations, as well as to elucidate the precise molecular mechanisms involved.

## Figures and Tables

**Table 1:** Kidney/Tibia Ratio morphometry measurements in the *E. tirucalli* treated (n = 8) and control animals (n = 8).

Group\Parameters	Kidney Weight (mg)	Tibia Length (cm)	Kidney/Tibia Ratio (mg/cm)
Control	1353 ± 22.97	4.36 ± 0.02	310.2 ± 4.29
*E. tirucalli*	1313 ± 11.04	4.36 ± 0.02	301.2 ± 2.99

## Data Availability

Data are contained within the article.

## References

[1] Bonow C.T., Ceolin T., Lopes C.V., Graciela J., Zillmer V., Rosiely N., Vargas C., Heck R. (2020). Plantas Medicinais Utilizadas na Auto atenção por Pessoas com Câncer em Cuidado Paliativo. Texto Contexto Enferm..

[2] Schmelzer G.H., Gurib-Fakim A., Arroo R., Bosch C.H., de Ruijter A., Simmonds M.S.J., Lemmens R.H.M.J., Oyen L.P.A. Medicinal plants 1 Plant Resources of Tropical Africa. https://www.estantevirtual.com.br/livro/dicionario-das-plantas-uteis-do-brasil-1KP-0558-000.

[3] Cruz L.G. (1964). Dicionário das Plantas Úteis do Brasil.

[4] Dantas I.C. (2007). O Raizeiro.

[5] Wal A., Wal P., Gupta N., Vishnoi G., Srivastava R.S. (2013). Medicinal Value of *Euphorbia tirucalli*. Int. J. Pharm. Biol. Arch..

[6] Mali P.Y., Panchal S.S. (2017). *Euphorbia tirucalli* L.: Review on morphology, medicinal uses, phytochemistry and pharmacological activities. Asian Pac. J. Trop. Biomed..

[7] Dutra R.C., Souza P., Bento A.F., Marcon R., Bicca M.A., Pianowski L.F., Calixto J.B. (2012). Euphol prevents experimental autoimmune encephalomyelitis in mice: Evidence for the underlying mechanisms. Biochem. Pharmacol..

[8] Cataluña P., Rates S.M.K. (1999). The traditional use of the latex from *Euphorbia tirucalli* Linnaeus (Euphorbiaceae) in the treatment of cancer in south Brazil. Acta Hortic..

[9] Goel G., Makkar H.P.S., Francis G., Becker K. (2007). Phorbol esters: Structure, biological activity, and toxicity in animals. Int. J. Toxicol..

[10] Newton A.C. (2018). Protein kinase C as a tumor suppressor. Semin. Cancer Biol..

[11] Binckley S., Zahra F. (2022). Euphorbia tirucalli Toxicity.

[12] Rodrigues M.L., Gomes A.J., Funez M.I., Marques S., Lunardi C.N. (2022). *Euphorbia tirucalli* latex loaded polymer nanoparticles: Synthesis, characterization, in vitro release and in vivo antinociceptive action. PLoS ONE.

[13] Bessa G., Melo-Reis P., Araújo L., Mrué F., Freitas G., Brandão M., Silva Júnior N. (2015). Angiogenic activity of latex from *Euphorbia tirucalli* Linnaeus 1753 (Plantae, Euphorbiaceae). Braz. J. Biol..

[14] Abdel-Aty A.M., Hamed M.B., Salama W.H., Ali M.M., Fahmy A.S., Mohamed S.A. (2019). *Ficus carica*, *Ficus sycomorus* and *Euphorbia tirucalli* latex extracts: Phytochemical screening, antioxidant and cytotoxic properties. Biocatal. Agric. Biotechnol..

[15] Martins C.G., Appel M.H., Coutinho D.S.S., Soares I.P., Fischer S., de Oliveira B.C., Fachi M.M., Pontarolo R., Bonatto S.J.R., Fernandes L.C. (2020). Consumption of latex from *Euphorbia tirucalli* L. promotes a reduction of tumor growth and cachexia, and immunomodulation in Walker 256 tumor-bearing rats. J. Ethnopharmacol..

[16] Santana S.S., Gennari-Cardoso M.L., Carvalho F.C., Roque-Barreira M.C., Santiago A.S., Alvim F.C., Pirovani C.P. (2014). Eutirucallin, a RIP-2 type lectin from the latex of *Euphorbia tirucalli* L. presents proinflammatory properties. PLoS ONE.

[17] Avelar B.A., Lelis F.J.N., Avelar R.S., Weber M., Souza-Fagundes E.M., Lopes M.T.P., Martins-Filho O.A., Brito-Melo G.E.A. (2011). The crude latex of *Euphorbia tirucalli* modulates the cytokine response of leukocytes, especially CD4+ T lymphocytes. Braz. J. Pharmacogn..

[18] Neiva L. (1968). A cura do Câncer pelo Aveloz.

[19] Costa L.S. (2011). Estudo do uso do Aveloz (Euphorbia tirucalli) no Tratamento de Doenças Humanas: Uma Revisão.

[20] FDA (2005). Estimating the Safe Starting Dose in Clinical Trials for Therapeutics in Adult Healthy Volunteers.

[21] Reagan-Shaw S., Nihal M., Ahmad N. (2008). Dose translation from animal to human studies revisited. FASEB J..

[22] Lima I.L.B., Bose P., Rodrigues A., Bergamaschi C.T., Campos R.R., Hirata A.E., Tufik S., Xylaras B.P., Visniauskas B., Chagas J.R. (2014). Chronic sleep restriction during pregnancy—Repercussion on cardiovascular and renal functioning of male offspring. PLoS ONE.

[23] Fuhr J., Kaczmarczyk J., Kruttgen C.D. (1955). A simple colorimetric method of inulin determination in renal clearance studies on metabolically normal subjects and diabetics. Klin. Wochenschr..

[24] Saud A., Luiz R., Paula A., Müller C.R., Visoná I., Reinecke N.L., Silva W.H., Aparecida M., Razvickas C.V., Casarini D.E. (2021). Resistance exercise training ameliorates chronic kidney disease outcomes in a 5/6 nephrectomy model. Life Sci..

[25] Bradford M.M. (1976). A rapid and sensitive method for the quantitation of microgram quantities of protein utilizing the principle of protein-dye binding. Anal. Biochem..

[26] Ozenirler S., Erkan G., Degertekin C.K., Ercin U., Cengiz M., Bilgihan A., Yilmaz G., Akyol G. (2014). The relationship between advanced oxidation protein products (AOPP) and biochemical and histopathological findings in patients with nonalcoholic steatohepatitis. J. Dig. Dis..

[27] Bradley P.P., Christensen R.D., Rothstein G. (1982). Cellular and extracellular myeloperoxidase in pyogenic inflammation. Blood.

[28] Dias A.T., Rodrigues B.P., Porto M.L., Gava A.L., Balarini C.M., Freitas F.P.S., Palomino Z., Casarini D.E., Campagnaro B.P., Pereira T.M.C. (2014). Sildenafil ameliorates oxidative stress and DNA damage in the stenotic kidneys in mice with renovascular hypertension. J. Transl. Med..

[29] Folkmann J.K., Loft S., Moller P. (2007). Oxidatively damaged DNA in aging dyslipidemic ApoE−/− and wild-type mice. Mutagenesis.

[30] Tonini C., Campagnaro B., Louro L., Pereira T., Vasquez E., Meyrelles S. (2013). Effects of aging and hypercholesterolemia on oxidative stress and DNA damage in bone marrow mononuclear cells in apolipoprotein E-deficient mice. Int. J. Mol. Sci..

[31] Twaij B.M., Hasan M.N. (2022). Bioactive secondary metabolites from plant sources: Types, synthesis, and their therapeutic uses. Int. J. Plant Biol..

[32] Gounden V., Bhatt H., Jialal I. (2023). Renal Function Tests.

[33] Meltzer J.S. (2013). Renal Physiology.

[34] Johns E.J., Ahmeda A.F. (2014). Renal circulation. Ref. Modul. Biomed. Sci..

[35] Butterworth J.F., Mackey D.C., Butterworth J.F., Mackey D.C., Wasnick J.D. (2001). Pharmacology of anesthetic agents. Morgan & Mikhail’s Clinical Anesthesiology.

[36] Piper S.N., Suttner S.W., Maleck W.H., Boldt J. (2004). Comparison of propofol and thiopental for induction of anesthesia in patients with coronary artery disease. J. Cardiothorac. Vasc. Anesth..

[37] Verbalis J.G. (2006). Disorders of body water homeostasis. Best Pract. Res. Clin. Endocrinol. Metab..

[38] Förstermann U., Sessa W.C. (2012). Nitric oxide synthases: Regulation and function. Eur. Heart J..

[39] Khalil R. (2013). Protein kinase C inhibitors as modulators of vascular function and their application in vascular disease. Pharmaceuticals.

[40] Ringvold H.C., Khalil R.A. (2017). Protein kinase C as regulator of vascular smooth muscle function and potential target in vascular disorders. Adv. Pharmacol..

[41] Boubaker K., Ounissi M., Brahmi N., Goucha R., Hedri H., Abdellah T., El Younsi F., Maiz H., Kheder A. (2013). Acute renal failure by ingestion of *Euphorbia paralias*. Saudi J. Kidney Dis. Transpl..

[42] Al-Yousef H.M., Alqahtani A.S., Ghani A.S.A., El-Toumy S.A., El-Dougdoug W.I.A., Hassan W.H.B., Hassan H.M. (2020). Nephroprotective, cytotoxic and antioxidant activities of *Euphorbia paralias*. Saudi J. Biol. Sci..

[43] Habib S.L. (2018). Kidney atrophy vs hypertrophy in diabetes: Which cells are involved?. Cell Cycle.

[44] Zhang C., Shao M., Yang H., Chen L., Yu L., Xiao J., Tian H., Zhang F., Cheng P., Jin L. (2013). Attenuation of hyperlipidemia- and diabetes-induced early-stage apoptosis and late-stage renal dysfunction via administration of fibroblast growth factor-21 is associated with suppression of renal inflammation. PLoS ONE.

[45] Verma S., Singh P., Khurana S., Ganguly N.K., Kukreti R., Saso L., Rana D.S., Taneja V., Bhargava V. (2021). Implications of oxidative stress in chronic kidney disease: A review on current concepts and therapies. Kidney Res. Clin. Pract..

[46] Gyurászová M., Gurecká R., Bábíčková J., Tóthová Ľ. (2020). Oxidative stress in the pathophysiology of kidney disease: Implications for noninvasive monitoring and identification of biomarkers. Oxid. Med. Cell. Longev..

[47] Meyrelles S.S., Peotta V.A., Pereira T.M., Vasquez E.C. (2011). Endothelial dysfunction in the apolipoprotein E-deficient mouse: Insights into the influence of diet, gender and aging. Lipids Health Dis..

[48] Basile D.P., Leonard E.C., Beal A.G., Schleuter D., Friedrich J. (2012). Persistent oxidative stress following renal ischemia-reperfusion injury increases ANG II hemodynamic and fibrotic activity. Am. J. Physiol. Renal Physiol..

[49] Podkowińska A., Formanowicz D. (2020). Chronic kidney disease as oxidative stress- and inflammatory-mediated cardiovascular disease. Antioxidants.

[50] Juan C.A., Pérez de la Lastra J.M., Plou F.J., Pérez-Lebeña E. (2021). The chemistry of reactive oxygen species (ROS) revisited: Outlining their role in biological macromolecules (DNA, lipids and proteins) and induced pathologies. Int. J. Mol. Sci..

[51] Basu P., Hornung R.S., Averitt D.L., Maier C. (2019). *Euphorbia bicolor* (Euphorbiaceae) latex extract reduces inflammatory cytokines and oxidative stress in a rat model of orofacial pain. Oxid. Med. Cell. Longev..

[52] Tsumbu C.N., Deby-Dupont G., Tits M., Angenot L., Frederich M., Kohnen S., Mouithys-Mickalad A., Serteyn D., Franck T. (2012). Polyphenol content and modulatory activities of some tropical dietary plant extracts on the oxidant activities of neutrophils and myeloperoxidase. Int. J. Mol. Sci..

[53] Ratliff B.B., Abdulmahdi W., Pawar R., Wolin M.S. (2016). Oxidant mechanisms in renal injury and disease. Antioxid. Redox Signal..

[54] Andrés C.M.C., Pérez de la Lastra J.M., Juan C.A., Plou F.J., Pérez-Lebeña E. (2023). Superoxide anion chemistry—Its role at the core of the innate immunity. Int. J. Mol. Sci..

[55] Kaushal G.P., Chandrashekar K., Juncos L.A. (2019). Molecular interactions between reactive oxygen species and autophagy in kidney disease. Int. J. Mol. Sci..

[56] Freitas F.P., Porto M.L., Tranhago C.P., Piontkowski R., Miguel E.C., Miguel T.B., Martins J.L., Nascimento K.S., Balarini C.M., Cavada B.S. (2015). *Dioclea violacea* lectin ameliorates oxidative stress and renal dysfunction in an experimental model of acute kidney injury. Am. J. Transl. Res..

[57] Griner E.M., Kazanietz M.G. (2007). Protein kinase C and other diacylglycerol effectors in cancer. Nat. Rev. Cancer.

[58] Wu-Zhang A.X., Newton A.C. (2013). Protein kinase C pharmacology: Refining the toolbox. Biochem. J..

[59] Griendling K.K., Camargo L.L., Rios F.J., Alves-Lopes R., Montezano A.C., Touyz R.M. (2021). Oxidative stress and hypertension. Circ. Res..

[60] Gopalakrishna R., Jaken S. (2000). Protein kinase C signaling and oxidative stress. Free Radic. Biol. Med..

[61] Roberts A.C., Porter K.E. (2013). Cellular and molecular mechanisms of endothelial dysfunction in diabetes. Diabetes Vasc. Dis. Res..

[62] Li X.-J., Suo P., Wang Y.-N., Zou L., Nie X.-L., Zhao Y.-Y., Miao H. (2024). Arachidonic acid metabolism as a therapeutic target in AKI-to-CKD transition. Front. Pharmacol..

[63] Li J., Gobe G. (2006). Protein kinase C activation and its role in kidney disease (review article). Nephrology.

[64] Nowak G., Bakajsova D. (2012). Protein kinase C-α activation promotes recovery of mitochondrial function and cell survival following oxidant injury in renal cells. Am. J. Physiol. Renal Physiol..

[65] Lee S.-J., Kim D.-C., Choi B.-H., Ha H., Kim K.-T. (2006). Regulation of p53 by activated protein kinase C-δ during nitric oxide-induced dopaminergic cell death. J. Biol. Chem..

